# Supplementary figures and images for: Endosomal structure and APP biology are not altered in a preclinical mouse cellular model of Down syndrome
Source: PLoS One. 2022 May 11;17(5):e0262558. doi: 10.1371/journal.pone.0262558 (PMC9094519; doi:10.1371/journal.pone.0262558)

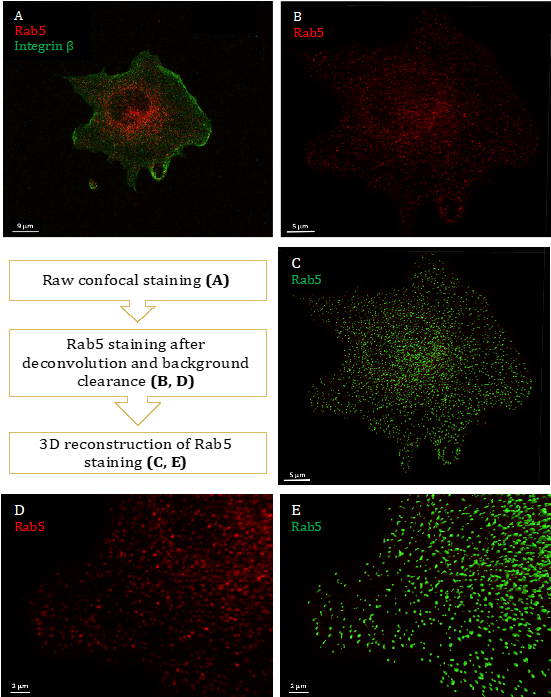

Supplement: S1 Fig — A) WT MEF stained for Integrinβ (cell membrane, green) and RAB5 (endosomes, red). B, D) Endosomal staining after deconvolution and background clearance C, E) 3D reconstruction of endosomal staining. Deconvolution and 3D reconstruction to accurately quantify the volume of endosomes. Z-stacks of each cell were taken with 150 nm interval between slices and fixed voxel volume (x = 50 nm, y = 50 nm, z = 150 nm) on confocal microscope LSM880. Each stack was deconvolved using Huygens software to improve image signal to noise and resolution. ImageJ software was used to remove the background with a macro written by Dr Dale Moulding. Imaris software was used to reconstruct the deconvolved staining in 3D. The area of Integrinβ was used to create a mask to define cellular volume. (TIF) [file pone.0262558.s001.tif]

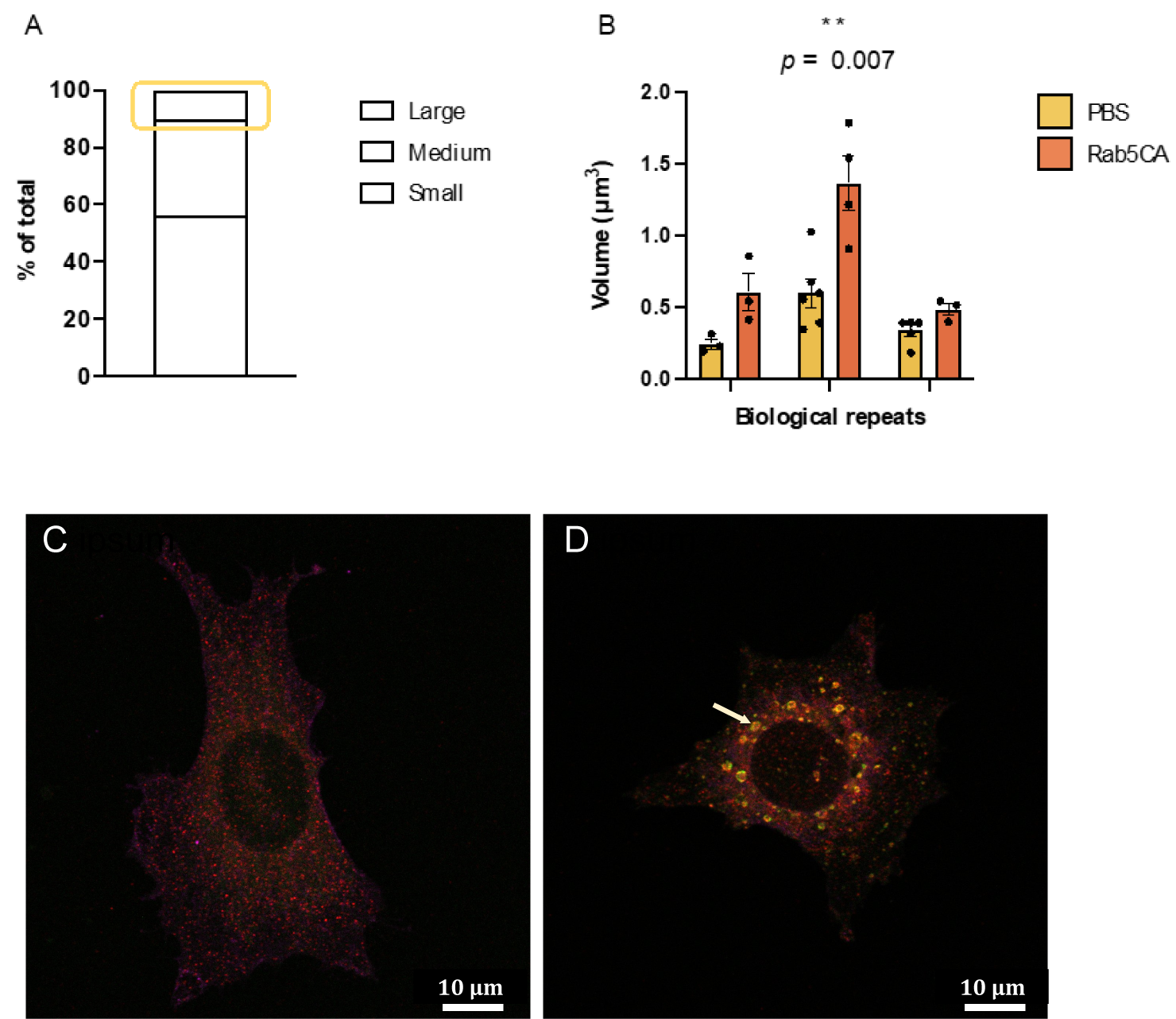

Supplement: S2 Fig — A) The normal distribution of endosomal size in WT MEFs transfected with PBS was determined to define the parameters for classification of “large” endosomes (small: endosomes in the 0–50th percentile, medium: endosomes in the 50–90th percentile, large: endosomes in the >90thpercentile). B) A nested t-test showed that ‘large’ endosomes in cells transfected with RAB5CA had a significantly higher volume than the endosomes in cells transfected with PBS (p = 0.007, N = 3 of biological repeats). The dots indicate the average volume of the ‘large’ endosomes in one cell imaged (technical repeat). Error bars = SEM. C, D) Representative images of WT MEFs transfected with PBS (C) or RAB5CA (D), endosomes labelled with RAB5 antibody (red), the RAB5CA plasmid is GFP-tagged (green); enlarged endosome indicated with (white arrow). (TIF) [file pone.0262558.s002.tif]

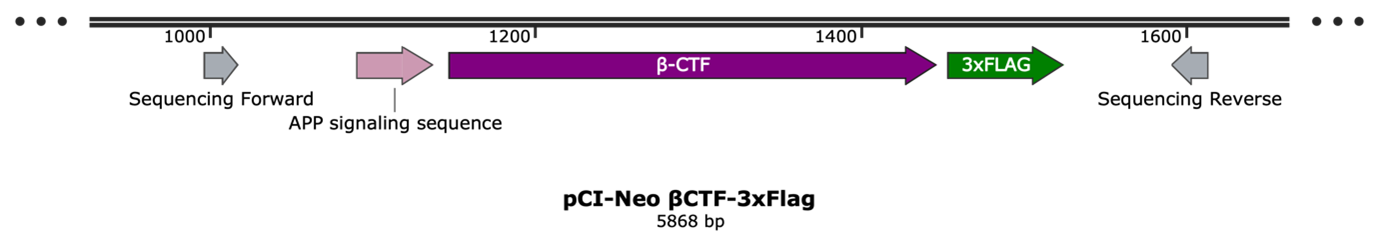

Supplement: S3 Fig — The APP signalling sequence was inserted in a pCI-neo plasmid followed by the β-CTF fragment of APP and by a 3xFLAG sequence. The primers used for sequencing the insert (sequencing forward and reverse) are also shown. (TIF) [file pone.0262558.s003.tif]
