# Supplementary material for: Endosomal structure and APP biology are not altered in a preclinical mouse cellular model of Down syndrome
Source: PLoS One. 2022 May 11;17(5):e0262558. doi: 10.1371/journal.pone.0262558 (PMC9094519; doi:10.1371/journal.pone.0262558)
Supplement: S4 Fig — Macro designed by Dr Dale Moulding to smooth the cell surface and clear its outside in 3D, enabling accurate quantification of the volume of the cell and of the number and volume of endosomes. (PDF) [file pone.0262558.s004.pdf]

S4 Fig Custom ImageJ Macro. Macro designed by Dr Dale Moulding to smooth the cell surface and clear its outside in 3D, enabling accurate quantification of the volume of the cell and of the number and volume of endosomes.

Macro:

```
rename("Orig");
run("Split Channels");
selectWindow("C2-Orig");
run("Gaussian Blur 3D...", "x=2 y=2 z=2");
setAutoThreshold("Huang dark stack");
//run("Threshold...");
run("Convert to Mask", "method=Huang background=Dark");
run("Analyze Particles...", "size=3-Infinity show=Masks stack");
selectWindow("Mask of C2-Orig");
run("16-bit");
run("Divide...", "value=255 stack");
imageCalculator("Multiply create stack", "C1-Orig","Mask of C2-Orig");
```
